# Exosomes in the Thymus: Antigen Transfer and Vesicles

**DOI:** 10.3389/fimmu.2015.00366

**Published:** 2015-07-20

**Authors:** Gabriel Skogberg, Esbjörn Telemo, Olov Ekwall

**Affiliations:** ^1^Department of Rheumatology and Inflammation Research, Institute of Medicine, The Sahlgrenska Academy, Gothenburg University, Gothenburg, Sweden; ^2^Department of Pediatrics, Institute of Clinical Sciences, The Sahlgrenska Academy, Gothenburg University, Gothenburg, Sweden

**Keywords:** exosome, thymic epithelial cell, tolerance, tissue-restricted antigen, miRNA

## Abstract

Thymocytes go through several steps of maturation and selection in the thymus in order to form a functional pool of effector T-cells and regulatory T-cells in the periphery. Close interactions between thymocytes, thymic epithelial cells, and dendritic cells are of vital importance for the maturation, selection, and lineage decision of the thymocytes. One important question that is still unanswered is how a relatively small epithelial cell population can present a vast array of self-antigens to the manifold larger population of developing thymocytes in this selection process. Here, we review and discuss the literature concerning antigen transfer from epithelial cells with a focus on exosomes. Exosomes are nano-sized vesicles released from a cell into the extracellular space. These vesicles can carry proteins, microRNAs, and mRNAs between cells and are thus able to participate in intercellular communication. Exosomes have been shown to be produced by thymic epithelial cells and to carry tissue-restricted antigens and MHC molecules, which may enable them to participate in the thymocyte selection process.

## Introduction

Exosomes are small (30–100 nm) vesicles released by cells into the extracellular space. They are a subgroup of extracellular vesicles (EVs) formed by inward budding of membranes in the late endosomes, thus creating a multi-vesicular body (MVB), which may dock to the outer membrane of the cell and release its content of exosomes (1, 2). In hindsight, the recycling of the transferrin receptor back to the cell membrane from an endocytic route in developing erythrocytes was the original description of exosomes in 1983 (3). One particularly striking finding, which in recent years has heavily influenced exosome research, was the identification of microRNA (miRNA) and mRNA in exosomes (4). Exosomal transfer of functional miRNA and mRNA has been demonstrated to result in regulation of gene expression through miRNA as well as translation of proteins through mRNA in recipient cells (5, 6). Exosomes are abundant in, and fairly easy to purify from, bodily fluids such as saliva (7), peripheral blood (8), bronco alveolar lavage fluid (9), and urine (10, 11), and one area of extensive research is the use of exosomes as diagnostic biomarkers for various pathologic conditions (12).

Exosomes have also shown therapeutic promise, e.g., in a study by Zitvogel and co-workers it was demonstrated that tumor peptide-pulsed dendritic cells (DCs) released exosomes that carried MHC I and II as well as co-stimulatory molecules and that these exosomes primed cytotoxic T-cells and suppressed the growth of established tumors (13). Native antigens on tumor-derived exosomes can be taken up by DCs and cross-presented to tumor-specific cytotoxic T-cells (14).

Other studies have revealed that exosomes are capable of presenting antigens to T-cells. In 1996, Raposo et al. (15) reported that both human and murine B-cell derived exosomes could induce an antigen-specific MHC II-restricted T-cell response. Exosome-like structures, named tolerosomes, are released from epithelial cells of the small intestine and have been shown to induce specific tolerance to fed antigens (16). As with the B-cell derived exosomes, tolerosomes seem to deliver antigens in a MHC dependent manner (17).

The thymus is the organ responsible for the establishment of an immune competent but yet self-tolerant T-cell population. While the extreme diversity of the mature effector T-cell specificities is a prerequisite for an effective defense against invading pathogens/infectious agents, the negative selection of self-reactive effector T-cells and the positive selection of T regulatory cells ensure tolerance to self-structures in order to avoid autoimmunity. To ensure a self-tolerant peripheral population, thymocytes are selected against a comprehensive set of self-antigens (18) of which many are produced and presented by medullary thymic epithelial cells (mTECs) (19) under control of the autoimmune regulator (AIRE) (20). The importance of a functional thymic antigen expression has been validated in models where even a defect expression of a single antigen may lead to development of peripheral organ-specific autoimmunity (21). Of equal importance is the selection of regulatory T-cells (Tregs) within the thymus, foremost from clones with a somewhat elevated TCR avidity for self-antigens (22). Proper thymic Treg development is also dependent on AIRE, and in particular for the functionally important Tregs that are induced during the perinatal period (23).

Since thymocytes outnumber TECs by several orders of magnitude, and the fact that each individual TEC only expresses a small subgroup of self-antigens (18, 24), a possible dissemination of antigen from TECs has been discussed in order to aid individual TECs to cover an extended volume of the thymic microenvironment (25). This would increase the number of possible antigen–thymocyte interactions and allow additional impact of the thymic DC populations. Antigen transfer could potentially occur by different means such as apoptotic bodies, nanotubes, and/or exosomes. Here, we review the current literature and argue that thymic exosomes have a potential role in T-cell maturation and selection.

## Antigen Transfer and Indirect Antigen Presentation in the Thymus

Clearly, direct cell–cell contacts in the thymus are pivotal for the development of a functional T-cell population (26). The addition of antigen transfer between thymic cell populations could, however, optimize thymic cell communication by making antigens more available to the pool of developing thymocytes. Through the last two decades, a number of studies performed under different experimental conditions have demonstrated the transfer of antigens from TECs to DCs. Already in 1994, Kyewski and co-workers observed intercellular transfer of Eα_52–68_ (a-chain of the MHC class II allele I-E^d^, amino acids 52–68) from TECs to thymic DCs in a unidirectional fashion (27). They proposed that this mechanism “may enhance the efficacy of tolerance induction by spreading self-antigens” (27). Subsequent experiments with OVA-specific TCR-transgenic mice (RIP-mOVA model) revealed that transfer of antigen to and presentation by hematopoietic cells also applies to MHC class I-restricted epitopes, since it resulted in deletion of both MHC class I- and MHC class II-restricted OVA-specific thymocytes when OVA was expressed only by mTECs (28). The identity of the hematopoietic cells in the RIP-mOVA study was unclear but further studies have revealed that deletion of CD11c+ cells using CD11c-Cre mice crossed with mice expressing diphtheria toxin under the control Rosa Locus containing a loxP-flanked STOP cassette results in increased frequencies of CD4+ thymocytes and increased CD4 T-cell infiltration into peripheral tissues (29). Likewise, Aschenbrenner and colleagues have observed that DCs capture mTEC-derived antigens and take part in deletional tolerance (30). This observation was strengthened by a study by Koble and Kyewski who demonstrated a presentation of TEC antigens by DCs. In their study, thymic but not peripheral DCs presented TEC-derived OVA to OVA-specific T-cells and were constitutively provided with mTEC-derived proteins (31). Further, unidirectional antigen transfer from mTEC to DCs was shown to also apply for native endogenous self-antigens *in vivo* (31). Non-redundant contribution of DCs and mTECs is further suggested based on simultaneous hematopoietic MHC class II deficiency and reduced MHC II expression on mTECs; this combination has an additive worsening effect on negative selection compared to either of the single deficiencies alone (32).

Also, the Treg formation seem to be dependent of DC-TEC cross-talk, which was elegantly demonstrated in a study on bone marrow chimeras in which CD28/B7 signaling was disrupted on either hematopoietic-derived antigen-presenting cells (APCs) or on TECs. The results showed that when B7 was restored in the hematopoietic-derived APCs this was enough to restore Treg numbers, hence hematopoietic-derived APCs and TECs can independently contribute to Treg development (33). The transfer of material in this study was unidirectional toward DCs, and the discussed mechanisms were primarily exosomes and apoptotic bodies. Transfer of material from TECs to DCs has also been demonstrated in the work by Hubert and co-workers in which OT-II restricted thymocytes were deleted by a soluble form of OVA that required presentation by bone marrow-derived cells (34). Using tetramer staining and transfer of bone marrow with ablated expression of MHC II, Taniguchi and co-workers found an abolished negative selection of T-cells specific for the AIRE-controlled self-antigen retinoid-binding protein (35). They concluded that intercellular transfer of the interphotoreceptor retinoid-binding protein peptide epitope of amino acids 277–290 from AIRE-expressing mTECs to bone marrow-derived APCs is important for negative selection of the investigated peptide. In re-aggregated thymic organ cultures, both the thymic epithelium and conventional DCs (as opposed to plasmacytoid DCs) have been shown capable of eliminating autoreactive CD4 thymocytes and to support natural Treg (nTreg) development on their own (36). In addition, Perry and co-workers recently reported that CD8 α+ DCs preferentially acquire and present AIRE-dependent antigens to developing Treg cells (37). They also showed that bone marrow-derived APCs and mTECs play non-overlapping roles in shaping of the T-cell receptor repertoire in terms of deletion and Treg selection (37).

## T-Cell Stimulation by Exosomes, With or Without DCs

**Figure 1:**
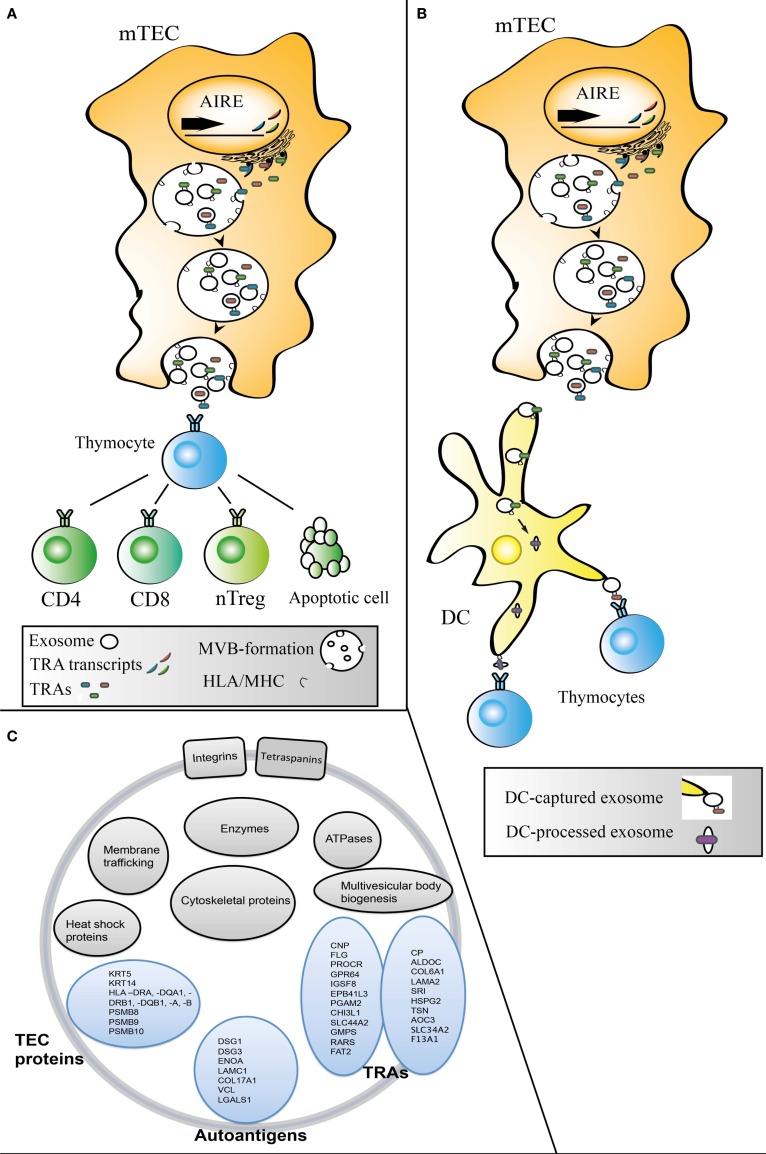
**Models for transfer of exosomal material from TECs to other thymic cell populations**. Transcription and translation of AIRE-dependent TRAs followed by TRA loading of exosomes, MVB fusion with the mTEC plasma membrane leading to exosome release from mTECs. The exosomes could then take an intercellular route from mTECs directly **(A)** to CD4+, CD8+, and developing nTregs and/or indirectly **(B)** via thymic DC or other APCs. **(C)** The proteome of thymic exosomes typically include exosomal markers, e.g., tetraspanins, TEC associated proteins, TRAs, and autoantigens (46, 48).

The capacity of exosomes to directly stimulate target cells has been debated. Some studies have suggested that there is an absolute need for DC presence for efficient exosomal stimulation of T-cells (38–40), while others have shown that exosomes are able to directly stimulate T-cells without any aid from DCs (15, 41–44). Models in which the efficiency of exosomal T-cell stimulation increases by DC presence have also been put forward (45). Interestingly, thymic exosomes carry ICAM-1 (46), which is both required for efficient T-cell responses (40) and involved in exosome binding to DCs (47). In addition to ICAM-1, thymus exosomes carry the opsonin MFGE8, which indicates that thymic exosomes would readily be engulfed by APCs such as DCs (46). TEC exosomes are also strongly positive for HLA-DR, which suggest a possibility that they contribute with antigens not only indirectly via, e.g., DCs but also directly to developing thymocytes (48). The presence of co-stimulatory molecules on exosomes may be important for their potential to affect the maturation of nTreg precursors (49). However, whether TEC exosomes that carry antigen presentation molecules and antigens need APCs or not to participate in thymocyte selection *in vivo* is not known. Possibly, thymic exosomes could take part in negative selection both directly by interacting with the thymocytes or indirectly by delivering antigens to APCs such as thymic DCs (Figures 1A,B).

## Characteristics of Thymic Exosomes

The first observation of exosome-like structures in thymic tissue was made by Wang and co-workers (50). These mouse thymic exosomes were characterized by high content of TGF-beta, CD9, and MHC II. In human beings, thymic exosomes were originally characterized with the use of explant cultures (46). The thymic exosomes shared features with exosomes from other sources, such as a size distribution of 30–100 nm for a majority of the vesicles, density peaking at 1.18–1.19 g/ml, which is less than the typical density of histone dense apoptotic bodies (51), and presence of typical exosomal proteins such as TSG101, CD9, CD81, and HLA-DR. Since these thymic exosomes were isolated from whole thymic tissue, the cellular source could not be determined, and the vesicles were most probably a mix of exosomes from different sources, e.g., thymocytes, TECs, and DCs. Even so, tissue-restricted antigens (TRAs), defined by protein-expression allowed in a maximum of five tissues in the human protein atlas (HPA) (52) were identified in the exosomes (2′,3′-cyclic-nucleotide 3′phosphodiesterase, reticulon 3, tropomyosin 3, and the GNAS protein), which suggest that a portion of the exosomes originates from the thymic epithelium (46). These four identified TRAs are possible candidates to participate in the selection/maturation processes within the human thymus. With the exception of one study that address thymic expression of 2′,3′-cyclic-nucleotide 3′phosphodiesterase (53), the four TRAs are hitherto unaddressed in thymic research. Interestingly, 2′, 3′-cyclic-nucleotide 3′phosphodiesterase is recognized by IgG autoantibodies in multiple sclerosis patients (54). In addition, tropomyosin 3 was suggested to be a candidate antigen in endometriosis (55).

Other traits seem to be specific for thymic exosomes compared to exosomes from other sources. One is the massive yield of ~1 mg of thymic exosomes per gram of thymic tissue grown in an explant culture (46). Other characteristics typical for thymic exosomes are the low expression of CD63 and the high expression of TSG101 on their surface (46). However, low levels of CD63 could have functional implications for thymic exosomes since it has been reported that siRNA mediated knockdown of the tetraspanin CD63 in a B-lymphoblastoid cell line (LCL) resulted in an increased CD4+ T-cell recognition as evaluated by IFN-γ production. The increase in T-cell response could not be explained by changes in antigen processing or MHC II-expression (56). Instead, equal amounts of exosomes from CD63^low^ LCL cells and control LCL cells stimulated the T-cells to comparable degrees, but the CD63^low^ LCL cells produced more exosomes, which in the end enhanced the total T-cell-stimulatory capacity of the CD63^low^ LCL cells.

Formal proof that TECs are able to produce exosomes was provided with the use of an approach in which primary cultures of TECs were established under selective conditions to eliminate the presence of thymocytes, DCs, fibroblasts, and peripherally produced exosomes (48, 57). The results showed that TECs produce exosomes and that these exosomes contained TRAs and a number of known autoantigens (48). Among the identified autoantigens in TEC exosomes were myelin basic protein (58), collagen type II (59), TITIN (60), heat shock protein 60 [connected with various autoimmune diseases (61)], transglutaminase 2 (62), desmoglein 1, and desmoglein 3 (63). In addition to autoantigens, previously reported mTEC-enriched TRAs were present in TEC exosomes, e.g., glutathione S-transferase M3 (GSTM3), LDL receptor, monocarboxylate transporter 4 (SLC16A3), mucins (MUC5B and MUC18), and myosin 1B (MYO1B) (48).

The observation that TEC exosomes have a higher fraction of proteins classified as TRAs (24%) compared to the fraction of TRAs in the cultured TECs (21%) could argue for a directed loading of TRAs into the exosomes (48).

Exosomes isolated directly from thymic tissue and exosomes isolated from TEC-cultures share a set of TRAs, such as 2′,3′-cyclic-nucleotide 3′ phosphodiesterase, which strengthens that thymic explant exosomes are partly of epithelial origin and that their TRA-content can be analyzed with whole thymic tissue as starting material. See Figure 1C for a schematic summary of the TEC-exosomal proteome. In addition, the presence of antigen-presenting molecules together with TRAs indicates that exosomes could transfer intact functional peptide-MHC complexes.

## RNA Transfer by Exosomes

**Figure 2:**
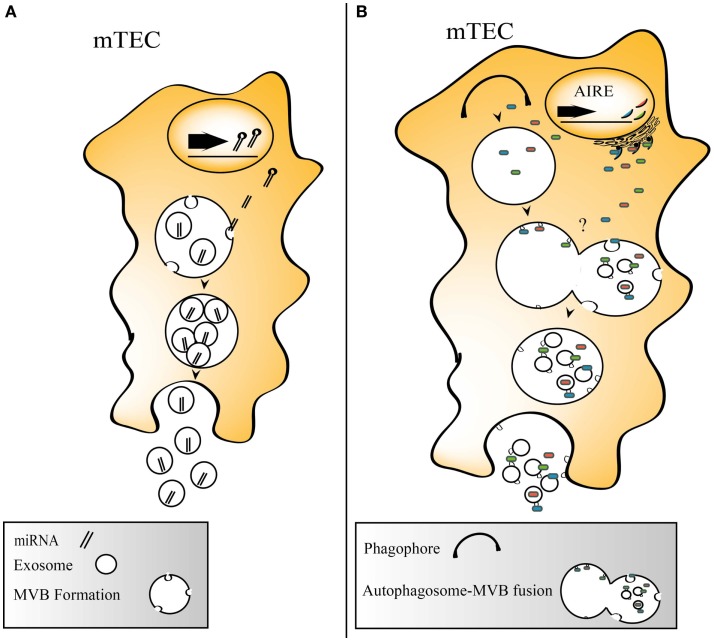
**(A)** Thymic exosomes contain miRNAs that could be taken up by adjacent cells. **(B)** Suggested model describing two principally different routes for TRA loading onto exosomes. TRAs could be engulfed by phagophores, which are processed to autophagosomes that fuse with MVBs where TRA carrying exosomes are formed. Alternatively, TRAs are directly directed into MVBs.

Exosomes have been increasingly recognized for their ability to transfer functional miRNAs and mRNAs between cells (4), and recently, it was shown that Tregs utilize exosomes for transfer of miRNA in order to functionally silence effector T-cells (64). The importance of miRNAs in the thymus has rendered an increased interest, and miRNAs have been shown to affect promiscuous gene expression under the influence of AIRE (65, 66), to be involved in thymic involution (67) and the maintenance of thymic epithelia (68). Thymic exosomes contain a number of miRNAs, and among them is the highly TEC enriched miRNA hsa-miR-149 (66). The role of thymic miRNAs has been thoroughly reviewed recently (69). Possible roles for miRNA sharing within the thymus by exosomes include control of TEC development and regulation of TRA expression. See Figure 2A for a schematic view of miRNA incorporation into TEC exosomes.

## Exosomal Biogenesis – Crossroads with Autophagy?

Autophagy may serve as a way to generate self-peptides from endogenously produced proteins that allows loading onto MHCII, and the constitutive autophagy process present in the thymus has been suggested to be involved in the negative selection and Treg formation (70). Thymic exosomes carry proteins involved in autophagy, such as autophagy related protein 7 (46). Two interesting observations are that a subgroup of TECs is rich in MVBs (71), and that at the same time, TECs have a constitutive autophagic activity (72). The simultaneous appearance of autophagic ultrastructure elements and multi-vesicular bodies in TECs support this notion (71). The intersection between autophagy and exosome formation is illustrated by the regulation of both autophagy and exosome biogenesis by GAIP interacting protein C terminus in pancreatic cancer cell lines (73). Exosomal release is also impaired in mouse embryonic fibroblasts lacking ATG12-ATG3, and immature autophagosomes are shown to fuse with MVBs in these cells (74). Whether this also occurs in TECs is so far unaddressed, but this route could potentially make antigens that are processed in the autophagic machinery available for export on exosomes and at the same time enhance the loading of endogenous antigens onto MHC class II molecules (Figure 2B).

## Concluding Remarks

Tissue-restricted antigen presentation within the thymic micromileus is pivotal to establish central tolerance. Antigen transfer between thymic cell populations, e.g., from mTECs to thymocytes and DCs is an established phenomenon that is poorly investigated from a mechanistic point of view. To get a more comprehensive understanding of central tolerance, the role of antigen transfer and the responsible vectors, e.g., exosomes, need to be studied and understood in more depth.

The abundant presence of TRA-containing exosomes in thymic tissue and the many observations of antigen transfer from mTECs to DCs lead us to speculate that this antigen transfer is, at least partly, mediated by exosomes. However, such a mechanism has not yet been formally proven. Although this review has been focused on possible antigen transfer by mTEC-derived exosomes to DCs and thymocytes, it is not excluded that also cTECs produce exosomes and that exosomes are shuttling antigens between different TEC populations. Other questions also remain regarding thymic exosomes that are equally important to answer in future studies; do exosomes exist *in vivo* in enough quantities to be biologically functional in the context of tolerance induction? If so, which is the primary route, direct interaction with thymocytes or indirect via APCs? What is the importance of exosomes for nTreg induction? Also, does miRNA content of the exosomes affect the development and maturation of thymic cells? Examples of experimental approaches that could be used to address these questions are TEC specific inhibition of the ESCRT machinery using the FOXN1-cre system or microinjection of thymic exosomes isolated from wild type mice into AIRE^−/−^ thymii. The outcome of this kind of experiments may give hints to whether a therapeutic use of tailor made exosomes to induce antigen-specific tolerance may be possible in the future.

## Conflict of Interest Statement

The authors declare that the research was conducted in the absence of any commercial or financial relationships that could be construed as a potential conflict of interest.
